# Factors related to tissue complications resulting from insulin therapy: a cross-sectional study*

**DOI:** 10.1590/1980-220X-REEUSP-2021-0249

**Published:** 2022-01-28

**Authors:** Wallison Pereira dos Santos, Mailson Marques de Sousa, Bernadete de Lourdes André Gouveia, Maria Júlia Guimarães Soares, Ana Maria de Almeida, Simone Helena dos Santos Oliveira

**Affiliations:** 1 Universidade Federal da Paraíba, Programa de Pós-graduação em Enfermagem, João Pessoa, PB, Brazil.; 2 Hospital Municipal Santa Isabel, João Pessoa, PB, Brazil.; 3 Universidade Federal de Campina Grande, Departamento de Enfermagem, Cuité, PB, Brazil.; 4 Universidade Federal da Paraíba, Departamento de Enfermagem Clínica, João Pessoa, PB, Brazil.; 5 Universidade de São Paulo, Escola de Enfermagem, Ribeirão Preto, SP, Brazil.

**Keywords:** Diabetes Mellitus, Insulin, Diabetes Complications, Needlestick Injuries, Nursing, Diabetes Mellitus, Insulina, Complicaciones de la Diabetes, Lesiones por Pinchazo de Aguja, Enfermería, Diabetes Mellitus, Insulina, Complicações do Diabetes, Ferimentos Penetrantes Produzidos por Agulha, Enfermagem

## Abstract

**Objective:**

To identify factors related to tissue complications resulting from insulin therapy.

**Method:**

This is a descriptive, cross-sectional study carried out in a capital of northeastern Brazil. A semi-structured form and an observation guide were applied to assess the performance of insulin preparation and administration techniques. Descriptive statistics, association test, and multivariate logistic regression were used for data analysis.

**Results:**

Most participants were female (74.2%), aged between 51 and 70 years (50.0%), and had nine to eleven years of education (36.7%). The presence of some type of local complication in 73.5% of the participants and the failure to rotate the injection sites in 82.3% are highlighted. Being single/widowed and not rotating insulin application sites were related to local complications and increased the chances of their occurrence by 3.51 and 6.70 times, respectively.

**Conclusion:**

Marital status and nonrotation of injection site were related to the increased chances of tissue complications resulting from insulin therapy.

## RESUMO

**Objetivo:**

Identificar os fatores relacionados à ocorrência de complicações teciduais decorrentes da insulinoterapia.

**Método:**

Estudo descritivo, transversal, realizado em capital do nordeste brasileiro. Aplicaram-se formulário semiestruturado e roteiro de observação para avaliação do desempenho das técnicas de preparo e administração de insulina. Estatística descritiva, teste de associação e regressão logística multivariada foram utilizadas para análise dos dados.

**Resultados:**

A maioria dos participantes era do sexo feminino (74,2%), com idades entre 51 e 70 anos (50,0%), e nove a onze anos de estudo (36,7%). Destaca-se a presença de algum tipo de complicação local em 73,5% dos participantes e a não realização do rodízio dos locais de injeção em 82,3%. Estar solteiro/viúvo e não realizar rodízio dos locais de aplicação da insulina relacionaram-se às complicações locais e aumentaram as chances de ocorrência destas em 3,51 e 6,70 vezes, respectivamente.

**Conclusão:**

O estado civil e a não realização de rodízio dos locais de aplicação mostraram-se relacionados ao aumento das chances de complicações teciduais decorrentes da insulinoterapia.

## RESUMEN

**Objetivo:**

Identificar factores relacionados con la aparición de complicaciones tisulares resultantes de la terapia con insulina.

**Método:**

Estudio descriptivo, transversal, realizado en la capital del noreste de Brasil. Se aplicó un formulario semiestructurado y un guión de observación para evaluar el desempeño de las técnicas de preparación y administración de insulina. Para el análisis de los datos se utilizó estadística descriptiva, prueba de asociación y regresión logística multivariante.

**Resultados:**

La mayoría de los participantes eran mujeres (74,2%), con edades comprendidas entre 51 y 70 años (50,0%) y con nueve a once años de escolaridad (36,7%). Se destaca la presencia de algún tipo de complicación local en el 73,5% de los participantes y mala rotación de los sitios de inyección en el 82,3%. Ser soltero/viudo y no rotar los sitios de aplicación de insulina se relacionaron con complicaciones locales y aumentaron la posibilidade de su ocurrencia en 3,51 y 6,70 veces, respectivamente.

**Conclusión:**

Se demostró que el estado civil y la mala rotación de los sitios de aplicación estaban relacionados con el aumento de las posibilidades de complicaciones tisulares como resultado de la terapia con insulina.

## INTRODUCTION

Diabetes Mellitus (DM) is characterized as a heterogeneous group of disorders causing dysfunction in the production/secretion/absorption of insulin, which results in hyperglycemia. Projections indicate that, for the year 2035, around 471 million people will be living with the disease worldwide^(1)^, highlighting that only in Latin America it is estimated that 40% of people are unaware of the diagnosis of DM. In 2017, there were 12.5 million Brazilians with DM, taking Brazil to the fourth place in number of cases of this metabolic disease^(2)^.

To control DM, adherence to pharmacological and non- pharmacological therapies is required. Insulin therapy is a complex treatment and does not depend exclusively on the user, The specificities of the materials used, availability for single use, as well as proper instructions for handling shall also be considered. Materials restriction can predispose the individual to their excessive reuse, and failure to rotate injection sites, as well as inadequate administration practices can lead to risks of infections and tissue trauma, in addition to inaccuracies in the dosage of insulin, which induce hyper- or hypoglycemia^(3–4)^.

Due to inadequate practice, the presence of tissue complications, such as hypertrophic lipodystrophies, hardened nodules, ecchymosis and abscesses, burning and itching resulting from failures in insulin preparation and administration can be highlighted. Therefore, ideal blood glucose levels are not reached and, as a consequence, local and systemic complications can be instituted due to non-adherence to the rotation of injection sites. Systemic complications, in their turn, are related to dosing errors and contamination of the materials used^(5–6)^.

Therefore, the institution of insulin therapy requires important measures in the process of dose preparation and subcutaneous, divided, daily administration of insulin. Care in insulin treatment, especially in administration, permeates a set of actions that shall be based on the interaction between professionals and users, since guidance and education shall be continuous, to avoid errors with negative outcomes in the insulinization process. Insulin therapy, practiced in collaboration among health professionals, users, and family members, is essential and beneficial in the achievement of glycemic control^(7)^.

Previous studies demonstrate the existence of gaps in the insulin preparation and administration process, inadequacies in the treatment and in the exposure of the user to unnecessary risks that lead to local and/or systemic complications^(8)^. Thus, the objective of this study was to identify factors related to tissue complications resulting from insulin therapy.

## METHOD

### Design of Study

A descriptive and cross-sectional study, developed according to the guidelines of the *Strengthening the Reporting of Observational Studies in Epidemiology* (STROBE), guided the report of this study.

### Local

Endocrinology Outpatient Clinic at Hospital Universitário Lauro Wanderley (*HULW*), located in João Pessoa, PB, Brazil.

### Population

Users of the above-mentioned clinic, diagnosed with DM, on use of insulin.

### Selection Criteria

The non-probabilistic, consecutive sample consisted of users diagnosed with type I and II DM, who met the inclusion criteria: age equal to or greater than 18 years and exclusive use of insulin therapy. Users in the first consultation were excluded, and the sample consisted of 136 participants.

### Data Collection

Participants were, through screening, invited to participate in the study, according to the list of users in outpatient care. When they decided to participate, they signed the Free and Informed Consent Term (FICF). It should be noted that there was no refusal from participants who met the selection criteria.

Data collection took place between October and December 2019, through the application of two instruments: the first consisted of a form to obtain sociodemographic and clinical data and the second corresponded to an observation guide to assess the performance of insulin preparation and administration techniques. It should be noted that information recording in the instruments was the investigator’s responsibility.

The form with 15 questions covered sociodemographic (age, sex, marital status, years of education, family income, and family arrangement), clinical (presence of symptoms and signs – lipohypertrophy, abscesses, bruises, nodules, ecchymoses, and infections – and time of diagnosis) variables, and supplies (type of insulin prescribed, number of daily doses, frequency of reuse of needle and syringe, injection site rotation, and type of material used).

The observation guide followed the recommendations of the Brazilian Society of Diabetes (SBD)^(8)^, based on ten questions about insulin preparation and eight about insulin administration. The eighteen items analyzed were: hand washing and drying; selection of materials for insulin preparation and application; homogenization of the insulin suspension; asepsis of the insulin bottle rubber; air aspiration with the needle cap on; needle cap removal and air injection into the insulin bottle; placement of the insulin bottle upside down; insulin aspiration up to the prescribed dose; elimination of air bubbles; needle protection until the moment of application; antisepsis with 70% alcohol at the application site; drying; performance of skinfold; needle injection with single, fast, firm, and light movement; continuous insertion of insulin into the tissue; maintenance of the injected needle for five seconds; gentle pressure on the site if there is bleeding. The items in the technique observation guide were categorized as performed (performed correctly), not performed (it was not performed), and inadequate (the item was performed, but incorrectly).

The data collection flow was organized in three stages: first – application of the sociodemographic form; second – performance of a clinical examination of the application sites, through the introductory methods of inspection and palpation; and third – development of the insulin administration technique, in a simulated environment, in which the step-by-step procedure was observed and recorded on the observation guide.

It should be noted that, for the third stage, a place to wash and dry hands, a trash can, and a sharps collector (descartex®) to disposal of the material were made available. At that time, the following materials were arranged on a table: 1-ml syringes (graduated in 100 international units), with fixed needles, syringes for removable needles, 4-mm, 6-mm, 8-mm and 12-mm removable needles, Neutral Protamine Hagedorn (NPH) insulin vials and regular insulin vials, insulin pens, pen needles, cotton, 70% alcohol, and insulin delivery prototype. Participants were invited to perform the technique, as they usually did in the home environment.

**Figure 1:**
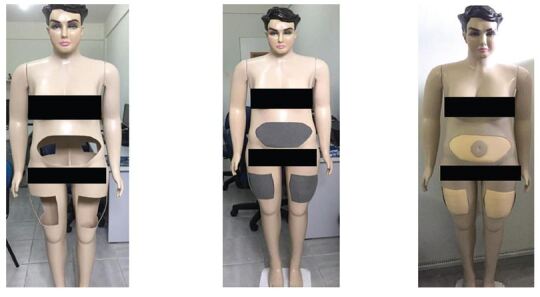
Prototype at different stages of manufacture for the development of the technique for insulin administration to the anterior aspect. João Pessoa, PB, 2020.

Source: Research image file, 2020.

To facilitate the simulation of insulin application, a prototype was built, according to recommendations^(9)^. To build the prototype, an obese female mannequin was used, in which cuts were made and foam laminate (D30) was inserted in the sites recommended for application and absorption of the administered insulin. The foam was covered with liquid silicone and a catalyst was used as a skin simulator allowing the performance of the skin fold. Finally, the ends of the synthetic skin were glued to the mannequin and the iron base for support, as shown in Figure 1.

### Data Analysis and Treatment

Descriptive and inferential statistics were used. Categorical variables were expressed as absolute and relative frequencies and numerical variables as dispersion measures (means). To observe associations among the variables under study, the chi-square and Fisher’s exact tests were used, in cases where the expected frequencies were less than five^(10)^.

To assess factors related to local complications of insulin therapy, multiple logistic regression models were built, considering the independent variables: sociodemographic (sex and marital status), supplies, and insulin administration technique (syringe type, needle diameter, and injection site rotation). As a dependent variable, local complications were established (bruises, ecchymosis, hypertrophies, infections, abscesses, and nodules), analyzed globally, as present or absent. To select the variables, the backward selection was performed, initially incorporating all the variables.

Next, the regression model was adjusted by the stepwise method. In the final model, only the variables that presented a significance level <0.20 were considered to identify factors related to complications resulting from insulin administration. Finally, the gross and adjusted Odds Ratios (OR) were estimated, with the respective Confidence Intervals (CI) and Wald tests, to verify the chances of complications occurring due to the participants’ characteristics or to the problems in the techniques of insulin preparation and/or administration. The analyses were carried out with the help of the R statistics software. The significance level of ≤0.05 was adopted for the study.

### Ethical Aspects

The project followed the regulations of Resolution no. 466/2012, being approved by the Research Ethics Committee of Hospital Universitário Lauro Wanderley, Universidade Federal da Paraíba, in 2019, with Opinion no. 3.457.517.

## RESULTS

A total of 136 individuals with Diabetes Mellitus participated in the study, with a predominance of female participants (74.2%), aged between 51 and 70 years (50.0%), married/in a common-law marriage (58.0%), family income of two or more minimum wages (51.4%), nine to eleven years of education (36.7%), and living with family members/caregivers (88.9%). In Table 1, the sociodemographic characteristics of the study participants are described.

**Table 1.** Sociodemographic characteristics of study participants in outpatient care – João Pessoa, PB, Brazil, 2020.

Variables		Absolute (n)	Relative (%)	p-value
Age (years)	20–30	11	8.0	<0.001
	31–40	15	11.0	
	41–50	19	13.9	
	51–60	29	21.3	
	61–70	39	28.6	
	71–80	17	12.5	
	≥81	6	4.4	
Sex	Male	35	25.7	<0.001
	Female	101	74.2	
Marital status	Single/Divorced/Widowed	57	41.9	0.0592
	Married/common-law marriage	79	58.0	
Family income (minimum wage)	<1	1	0.7	<0.001
	1	65	47.7	
	≥2	70	51.4	
Family arrangement	Live alone	15	11.0	<0.001
	Lives with family	121	88.9	
Years of studies	Never studied	10	7.3	<0.001
	1–4	39	28.6	
	5–8	25	18.3	
	9–11	50	36.7	
	≥12	12	8.8	
**TOTAL**		136	100.0	

Source: Survey data, 2020. Chi-square/Fisher’s exact test. *Current minimum wage in Brazil during the study period (2019): BRL 1,039.00.

In the simulation of insulin preparation, it was observed that many steps of the protocol were inadequately developed or were not performed. Among the steps that were completed, 56.6% of the participants gathered the material correctly, 84.5% followed the recommendation of not removing the needle and turning the bottle upside down, 79.4% aspirated insulin up to the prescribed dose, and 70.5% eliminated air bubbles after aspiration of the drug.

However, important steps in the preparation, such as the homogenization of insulin, were not performed by 24.2% of the participants and were inadequately performed by 39.7%. It was observed that about 80% did not carry out asepsis of the rubber in the insulin vial, 96% did not keep the needle cap when aspirating air up to the dose to be administered, and they did not keep the cap after aspiration of the drug until the administration. It should be noted that about 20% did not aspire the prescribed dose properly.

Regarding insulin administration technique, 66.9% of the participants did not perform antisepsis at the administration site and 94.1% did not keep the needle in the tissue after application, which can cause wastage of the dose, thus favoring uncontrolled glucose level, with the occurrence of hyperglycemia. In general, the procedure of insulin administration had important flaws of execution.

It was observed that the study participants had lived with DM, on average, for 14.54 years (±10.17), the most used type of insulin was NPH (44.1%), they did not adopt the practice of rotating injection sites (82.3%) and that the most frequently reported region for insulin administration was the abdomen (57.3%). It was identified that 39.7% used twice daily doses, 68.3% reused syringes and needles, and the average reuse corresponded to 5.24 times.

Regarding the specificities of the materials for insulin preparation and application, it was observed that the participants more frequently used syringes graduated with two International Units (IU) (58.8%), removable needles (89.7%) of 12 mm of diameter (44.8%).

Regarding symptoms and signs of complications from inadequate insulin application, clinical examination (inspection and palpation) showed a high frequency of tissue complications (73.5%), among which bruises, along with lipodystrophy (42.0%), were highlighted. Other events included pain, bruise and lipodystrophies (33.0%), lipodystrophies (16.0%), bruises (5.0%), isolated pain (3.0%), and burning and itching (1.0%).

When verifying the occurrence of complications from the practice of administration, statistical significance was obtained in relation to the non-adoption of injection site rotation (<0.001) and the use of the abdomen only as an injection region (<0.001). It was observed that among the participants who reused syringe and needle, the occurrence of complications was high (69%), especially the presence of bruises. It was also observed that the greatest number of complications related to tissue trauma occurred among those whose needle gauge was 12 mm (44%) (Table 2).

**Table 2.** Association of the characteristics of materials and insulin therapy practice with the occurrence of tissue complications – João Pessoa, PB, Brazil, 2020.

Variables		Local complications				p-value
		Yes		No		
		n	(%)	n	(%)	
**Inputs**					
	Regular	6	6.0	2	5.6	0.5576
	NPH	43	43.0	17	47.2	
Insulin type	Regular + NPH	32	32.0	9	25.0	
	Lantus	5	5.0	–	–	
	NR + Lantus	14	14.0	8	22.2
Type of syringe or pen	Graduation in IU	25	25.0	5	13.9	0.3905
	Graduation in IU	57	57.0	23	63.9	
	Graduated in ml	1	1.0	1	2.8	
	Application pen	17	17.0	7	19.4	
Needle type	Fixed	10	10.0	4	11.1	0.9477
	Removable	90	90.0	32	88.9	
Needle diameter	04 mm	11	11.0	5	13.9	0.9163
	05 mm	14	14.0	4	11.1	
	08 mm	31	31.0	10	27.8	
	12 mm	44	44.0	17	47.2	
**Insulin therapy**						
Daily prescribed doses	1	17	17.0	12	33,3	0.0519
	2	40	40,0	14	38,9	
	3	24	24,0	2	5.6	
	4	10	10.0	3	8,3	
	5	9	9,0	5	13.9	
Application sites	Arms	23	23.0	7	19.4	<0.001
	Abdomen	64	64.0	14	38.9	
	Thighs	2	2.0	2	5.6	
	All	11	11.0	13	36.1	
Carrying out rotation	Yes	11	11.0	13	36.1	<0.001
	No	89	89.0	23	63.9	
Reuse of syringes/needles	Yes	69	69.0	24	66.7	0.7962
	No	31	31.0	12	33.3	

Source: Survey data, 2020. Chi-square/Fisher’s exact test.

Table 3 shows the relationship of the variables sex, marital status, type of syringe, needle diameter and insulin injection site rotation with the occurrence of local complications. Among the five variables analyzed and that entered the multivariate regression model, only two remained significantly related to the emergence of tissue complications in the final model: marital status and application sites rotation. Single, divorced, or widowed insulin users had a 3.51 times increase in the chance of developing complications when compared to those who are married or in a common-law marriage. Similarly, the possibility of occurrence of complications increased by 6.70 times (95% CI 1.35 to 9.12) in those individuals who did not rotate insulin application sites.

**Table 3.** Logistic regression models of sociodemographic characteristics, materials, and insulin application technique related to the occurrence of local complications from insulin therapy – João Pessoa, PB, Brazil, 2020.

Variables	Model 1		Model 2		Model 3		Model 4	
	OR*	95% CI**	OR	95% CI	OR	95% CI	OR	95% CI
**Sex**								
Female	1.00	–	1.00	–	1.00	–		
Male	0.53	0.2–1.38	0.61	0.25–1.53	0,61	0,25–1,50		
**Marital status**								
Married/common-law marriage	1.00	–	1.00	–	1.00	–	1.00	–
Single/Divorced/ Widowed	**3.63**	1.32–9.96	**3.44**	1.28–9.24	**3,23**	1,23–8,50	**3,51**	1,35–9,12
**Syringe type**								
Graduation in IU	1.00	–						
Graduation in IU	0.45	0.14–1.47						
Graduation in ml	0.09	0.00–2.10						
Pen	0.68	0.13–3.46						
**Needle diameter**								
04 mm	1.00	–	1.00	–				
05 mm	2.31	0.40–13.23	2.04	0.39–10.54				
08 mm	2.54	0.55–11.74	2.33	0.54–9.98				
12 mm	2.08	0.43–10.14	1.68	0.44–6.37				
**Injection site rotation**								
Yes	1.00	–	1.00	–	1.00	–	1.00	–
No	**7.49**	2.47–22.71	**7.06**	2.42–20.60	**6.56**	2.31–18.62	**6.70**	1.35–9.12

Source: Survey data, 2020. Wald test (significance p < 0.20). *OR: Odds Ratio **CI: Confidence Interval.

When applying modeling to analyze the determination of these variables that achieved step 4 of the regression model, it was observed that both marital status and rotation are determining variables for the occurrence of tissue complications. It should be noted that despite the variables income, family arrangement and years of education have statistical significance (<0.001), they did not enter the logistic regression model, since they were excluded when Wald test was applied because they presented a high probability of not having an association with the dependent variable (tissue complications), that is, not showing significance p < 0.20 (Table 4).

**Table 4.** Multiple logistic regression with definition of determinant variables for the occurrence of complications – João Pessoa, PB, Brazil, 2020.

Variables	***β***	p-value	CI* (95%) for ***β***	
			Lower	Upper
Injection site rotation	1.9017	0.0003	0.891	2.996
Marital status	1.2561	0.0098	0.351	2.281

Source: Survey data, 2020.

## DISCUSSION

The results of this research describe predominantly elderly people who lived with DM for a long period, using insulin therapy as a treatment for glycemic control. Although administering insulin for more than six months, it was observed that most participants showed flaws in the practice of preparing and administering insulin, which resulted in local complications. It was identified that being married or living in a common-law marriage and not rotating the application sites are determining factors for the occurrence of these complications.

When considering insulin preparation and administration, the steps flaws were: inadequate homogenization of the insulin suspension; no insulin aspiration, according to the prescribed dose; no elimination of air bubbles; no antisepsis nor asepsis with alcohol; no introduction of the needle with a single, fast and firm movement; and no application of gentle pressure on the site, in cases of bleeding.

The inadequate preparation and administration can predispose to the emergence of local and systemic complications. Insulin is part of the list of five drugs that cause the most damage to adults and children, due to failures in use^(11)^. As it is a complex treatment, the steps of insulin preparation and administration must be strictly followed, to comply with the recommendations and guidelines of ministerial agencies, as well as those from qualified health professionals involved in the care of people with DM^(3)^.

The logistic regression model applied to identify the determining factors of complications resulting from insulin therapy revealed that the marital status variable was relevant for the occurrence of complications, showing that single, widowed, or divorced individuals have an increased chance of having local complications, leading to the inference that the support of the family and/or a significant person can be important for the adequate treatment in what regards insulin preparation and administration. Hence, the importance of including the user and family member in care.

In this regard, a study carried out in a state in the southern region of Brazil observed that having a partner can lead to a feeling of home resignification and characterization as an environment conducive to care, permeated by feelings of social support and protection expressed by the person who cares for and, in this way, stimulating the individual to cope with health problems and pathologies, in addition to preserving physical and cognitive conditions^(12)^. Thus, the fundamental role of the family, particularly of the partner, in supporting the care of individuals with DM is emphasized, to assist in the treatment of those who present difficulties or infeasibility in general self-care and in the administration of insulin.

Another relevant aspect is that most participants had nine to eleven years of education, which could influence the level of knowledge and understanding for the adoption of safe and appropriate practices related to insulin preparation and administration. However, no such impact was observed, considering that more than half of the participants had clinical symptoms and signs that were characteristic of inadequate practices in the procedures assessed.

Contrary to this result, the level of education has been considered one of the determining factors for the success of therapeutic adherence in the clinical management of DM. The low level of education is considered a factor that makes learning difficult and fragile, especially in relation to the understanding of therapeutic recommendations. It is assumed that higher levels of education are an adjuvant factor for understanding treatment approaches and, consequently, developing skills for less exposure to risk behaviors^(13)^.

Evidence in the literature highlights that there are gaps regarding the receipt of professional guidance on insulin therapy, a possible factor influencing inadequate preparation and administration practices^(14–15)^. It cannot be affirmed, based on the findings of this study, that this is the reality of the respondents; however, it is also not possible to rule it out. It is legitimate to ensure that the level of education is not the only factor influencing skills and it does not in itself ensure adequate practice, and that the cognition of those who participate in educational processes shall be considered, in addition to the capacity of the professional involved in the training, above all, of nurses who are in charge of educational actions.

When considering practices for the prevention and reduction of tissue complications, the adoption of injection sites rotation should be emphasized in health services and included in health education strategies for individuals who use insulin, since, in the present study, it was shown that most respondents did not rotate sites and only adopted the abdomen as a priority area for administration.

The non-adoption of rotation results in frequent subcutaneous administration in the same areas on a routine basis, favoring the occurrence of adverse events during the insulin administration process. Both the Ministry of Health and the SBD recommend the adoption of injection site rotation. It should be highlighted that for an adequate rotation, planning is required, with the identification of administration sites, signaling the last application point and ensuring a minimum distance of one and a half centimeters between one application and the other, avoiding repeating the site for at least twenty days^(16)^.

It is important to emphasize that for a safe rotation, an area shall be delimited for the application of all doses per week, thus exhausting the application in the delimited area and, later, another region shall be selected. It should be noted that these measures are able to reduce and/or prevent the emergence of tissue complications, such as lipodystrophies and abscesses^(8)^.

A study aiming at verifying the use of insulin and the factors related to its use points out that the interviewees report rotating injection sites, but they report a preference for application in the abdomen region, due to greater and better accessibility to the region, in addition to practicality^(17)^. Thus, it is important to discuss with individuals about the adequate absorption speed in the abdomen region, which is also obtained in other areas, such as the back of the arm and, finally, the thigh and upper gluteal region^(1)^.

Insulin preparation and administration errors with consequent tissue complications are frequent and deserve attention, as they cause discomfort and possible abandonment of the proposed therapy. In the present study, the most frequent complication was lipodystrophy, together with bruise, which is intrinsically still related to not performing rotation.

An international study, whose objective was to provide a reflection on clarity regarding the correct identification and treatment of lipodystrophy in individuals treated with insulin, points out that the frequent subcutaneous administration can favor the appearance of lipodystrophy, as well as other skin lesions, bruises. However, the most common lesion associated with insulin use is lipohypertrophy. It is evident that the consequences of this type of lipodystrophy are glycemic fluctuation, high glucose levels and, consequently, hyperglycemia^(5)^. Integumentary lesions resulting from insulin misuse can generate systemic complications, such as lack of glycemic control, requiring an adequate preparation and administration technique to carry out the insulin treatment.

This study has some limitations. The cross-sectional design makes it impossible to establish cause-effect relationships. The convenience sampling technique is subject to the risk of potential bias. Thus, the generalization of the findings shall be interpreted with caution. Another limitation to be recognized was the absence of a clinical parameter for hemoglobin A1c levels and the respective effects associated with the insulin preparation and administration technique. Future research shall be conducted with larger samples and in several locations, to assess the predictors of tissue complications. It is recognized that sociodemographic characteristics and health care access conditions directly impact adherence to satisfactory self-care.

As a contribution to nursing practice, this study presents relevant data for the development and adoption of educational strategies, focusing on the adequate preparation and administration of insulin, especially with regard to the adherence of individuals to the injection site rotation, as well as for the ability to judge on the reuse of syringes and needles, to reduce and even avoid the occurrence of local complications.

## CONCLUSION

The results of the study show individuals who have some type of complication at insulin administration sites. The practice of preparation and administration has limitations in several aspects, such as the number of reuses of syringes and needles, problems related to homogenization and correct aspiration of the prescribed dose, non-adoption of injection site rotation, and preference for applying in the abdominal region. It was identified that being married or living in a common-law marriage and rotating the application sites are determining factors for the reduction of these complications.
